# A finite difference study of radiative mixed convection MHD heat propagating Casson fluid past an accelerating porous plate including viscous dissipation and Joule heating effects

**DOI:** 10.1016/j.heliyon.2024.e28591

**Published:** 2024-03-27

**Authors:** B. Prabhakar Reddy, P.M. Matao, J.M. Sunzu

**Affiliations:** Department of Mathematics and Statistics, P. O. Box 338, The University of Dodoma, Tanzania

**Keywords:** MHD, Heat-generation, Buoyancy forces, Chemical reaction, Casson fluid, Viscous dissipation

## Abstract

A finite difference numerical simulation scrutiny is executed to evaluate the combined impacts of heat generation, buoyancy forces, viscous dissipation and Joule heating in unsteady hydro-magnetic mixed convective chemically reactive and radiative Casson fluid flowing along an exponentially accelerating permeable vertical plate engrossed in a porous media by considering ramp surface concentration and temperature. The dimensionless non-linear coupled PDEs describing the flow model are dealt numerically by adopting the competent implicit Crank-Nicolson finite difference procedure. The variance of velocity, temperature, and concentration distributions are exposed via graphical representations due to the dissimilarity of the flow restrained parameters. Computational outcomes of the skin-friction, Nusselt and the Sherwood numbers are portrayed in the tabular pattern. The final outcomes of the research exposed that the impacts of thermal radiation, viscous dissipation, and heat production parameters enlarges the temperature and velocity distributions. The fluid motion deflates for growing Casson parameter and magnetic field intensity. The rising chemical reaction parameter suppresses the concentration and velocity distributions. Very importantly it is distinguished that fluid momentum, temperature, and concentration are quicker in the instance of isothermal plate temperature than ramp wall temperature. This kind of research may find specific industrial and medical utilizations such as glass manufacturing, crude oil purification, lubrication, paper production, blood transport study in cardiovascular design, etc.

## 1 Introduction

Analyzing non-Newtonian fluid problems, particularly the Casson fluid problem, creates enormous applicability in pharmaceutical, chemical and cosmic industries, for example producing various chemicals, syrup, cleansers, juice, oil, gas, deodorizer, etc. The Casson fluid flow evaluation from the fluid dynamics perspective has been carried out by quite a few engineers, scientists and mathematicians. Casson [1] was the first investigation done on the Casson flow model to analyze for the pigment-oil suspension transport. The noteworthy investigations [[2], [3], [4], [5], [6], [7], [8]] discussed the conduct of Casson fluid transport under different circumstances. Mahanthesh et al. [9] interpreted Casson fluid's boundary-layer energy transfer flow inundated with dust atoms over three different geometries by considering heat producing and Cattaneo-Christov-type heat flux. Kataria and Patel [10] evaluated analytically heat absorption/generation impact on the MHD reacted Casson fluid flow above an accelerating exponentially permeable surface with ramp surface concentration and temperature. Rajput et al. [11] studied numerically solutal-Thermo non-Newtonian convective Casson radiated flow through vertical plate promulgated by Arrhenius-kinetics in the endurance of heat sink/source. Endalew [12] conducted an analytical investigation to evaluate heat-mass diffusion effects on Casson's unsteady flow with thermo-solutal boundary conditions over an oscillating vertical surface under thermo-solutal boundary conditions. Recently, Raghunath and Obulesu [13] and Raghunath et al. [14] highlighted the performance of Casson fluid flow under different effects with varied boundary conditions.

The consequences of chemical reaction and thermal radiation on heat and mass dispersion have been fascinating by several researchers on the grounds that their immense applicability in a collection of industries, scientific and engineering, such as solar power, high-temperature casting, thermo-nuclear fusion, propulsion devices for aircraft, missiles, food processing, chemical catalytic reactors, production of glassware, etc. Reddy and Rao [16] numerically evaluated radiation impact on hydro-magnetic convective unsteady fluid transport through the vertical porous surface in the persistence of thermo-diffusion, heat cause and Hall effect. Pramanik [17] presented the effects of radiation on heat transference Casson fluid flows across an permeable exponentially stretching surface. Kataria and Mittal [18] conceptualized a mathematical model to study radiation effects on gravity-driven optically dense convective nanofluid (NF) from an oscillating vertical plate. Seth et al. [19] utilized an analytical procedure to analyze the influent of chemical reactions on heat-absorbing hydro-magnetic natural convective flow due to an accelerated vertical porous surface amidst ramp-type temperature and concentration. Seth et al. [20] talked about the radiation impact on MHD heat-absorbing unsteady convective move through a speeding vertical plate with unstable ramp-type temperature and Hall current. The investigators [[21], [22], [23], [24], [25], [26]] exposed the chemical reaction and radiation impacts in different fluids under distant situations. Prabhakar Reddy [27] discussed the radiation effects on free-convective hydro-magnetic flow via an impulsively persuasive plate in the concurrent of Newtonian heating (NH) conditions and Hall current. Meenakumari et al. [28] reviewed the problem of unsteady radiative MHD reactive Williamson nanofluid on a permeable stretching surface. Prabhakar Reddy and Makinde [29] performed a numerical study on radiative and reactive hydro-magnetic heat absorbing flow during an impulsively travelling plate in the endurance of Hall impact with ramp surface temperature and concentration. Recently, Explorations [[30], [31], [32]] summarized the relevance of radiation and chemical reaction by engaging nanofluid and Casson nanofluid concealed by various conditions.

The above almost all cited investigations, they have deserted the viscous dissipation influence on the flow. However, it is incredibly precious to include the viscous dissipation impact in heat and mass transport analysis due to its influential applications in polymer manufacturing, instrumentation, tribology, lubrication, food processing, etc. Suneetha et al. [33] discussed dissipative heat impact on Magneto-hydrodynamic radiated free convection movement over an impulsively moving vertical surface. Hiteesh [34] scrutinized viscous-dissipation impact on radiated hydro-magnetic energy transfer flow due to the stretching plain surface in the survival of fluctuating energy-flux. Kishore et al. [35,36] determined the influent of dissipative heat on radiated MHD heat and mass diffusion under different conditions. Ibrahim and Reddy [37] studied radiating dissipative heat and mass transmission of MHD natural convection fluid passage with heat generation through a stretching surface. Reddy [38] inspected viscous dissipation impact on radiated convective MHD fluid with Hall current through a stretched vertical plate. Prabhakar Reddy [39] numerically scrutinized the influences of dissipation heat on MHD time-dependent free-convective incompressible fluid passage during a vertical plate of infinite length. Ibrahim et al. [40] evaluated the dissipation heat impact on reactive magneto-hydrodynamic mixed convective heat unveiling Casson nanofluid transport due to a non-linear permeable stretching surface. Gopal et al. [41] discussed the Joule and viscous dissipation impacts in reactive Casson flow with multiple slips across stretching sheet under the inclined magnetic intensity. Reddy et al. [42] studied viscous dissipation influence in the natural convective flow of unsteady Casson fluid from an oscillating surface. Mishra and Kumar [43] studied heat generation/absorption on dissipating Ag-water nanofluid past a Riga plate with suction velocity. Seth et al. [44] performed numerical analysis to inspect Joule and viscous dissipation influent on MHD Casson fluid transmitted above a flat vertical plate by considering thermo-diffusion and Newtonian heating with a chemical reaction of higher order. Prabhakar Reddy [45] numerically appraised the upshot of viscous dissipation on parabolic magneto-hydrodynamic unsteady thermally radiating fluid flow over an isothermal plate with chemical reaction. Mishra et al. [46,47] pondered impacts of viscous dissipation on the passage of MHD nanofluid by fetching stretching sheet and wedge along with other influences. Himanshu and Mishra [48] evaluated by using the models Cattaneo–Christov diffusion and Yamada–Ota the performance of hybrid nanofluid passage in a rotating disk. Meenakumari and Lakshminarayana [49] explained heat dissipation impacts on Jeffery nanofluid of second order slip flow in an inclined asymmetric porous. Prabhakar Reddy and Joseph [50] recently performed numerical analysis to study MHD heat-absorbing Casson dissipative fluid flowing with Newtonian heating through an oscillating vertical porous plate.

The motive of the current exploration is to research the influence of heat-generation and viscous dissipation on radiated magneto-hydrodynamic time-dependent reactive species Casson fluid transport across an exponentially escalating vertical porous plate by considering ramp surface temperature and concentration, as this problem is missing in the available literature. An explicit literature examination of the previously published influential works by Khalid et al. [4] and Kataria and Patel [5,10] considered the Casson fluid through the porous medium with varied boundary restrictions in their studies. But these studies did not consider the Casson parameter effect in the porosity term. Also, the term deriving energy dissipation by the influence of porous permeability was neglected by Gopal et al. [41] and Reddy et al. [42]. Therefore, our intense objective in this exploration is to address the above missing effects by broadening the previously published work of Kataria and Patel [10] by including the viscous dissipation and thermal radiation impacts, which demonstrates the novelty of current study. This model conception finds instantaneous applications in nuclear-waste repositories, catalytic reactors, fire dynamics in insulations, and manufacturing of pharmaceutical components, paints, synthetic lubricants and coal in water. The derived coupled non-linear governing set of PDEs of the problem is simplified numerically by utilizing the implicit Crank-Nicolson finite difference scheme. Furthermore, we compared the current and past existing results in the literature to confirm the accuracy and exactitude of the adopted numerical procedure and results. Aside from the described objective and aim of the study, this study provides the answers to the following research questions.

(i) How does the velocity, temperature and concentration distribution vary with respective the flow control parameters such as magnetic field, heat generation parameter, radiation parameter, Casson parameter, Eckert number, chemical reaction parameter, plate acceleration parameter, time, porosity parameter, thermal and solute buoyant forces?
(ii) What are the alterations in the coefficient of skin friction, Nusselt and Sherwood numbers due to the disparity in the parameters mentioned in (i)?

## 2 Mathematical description

**Fig. 1:**
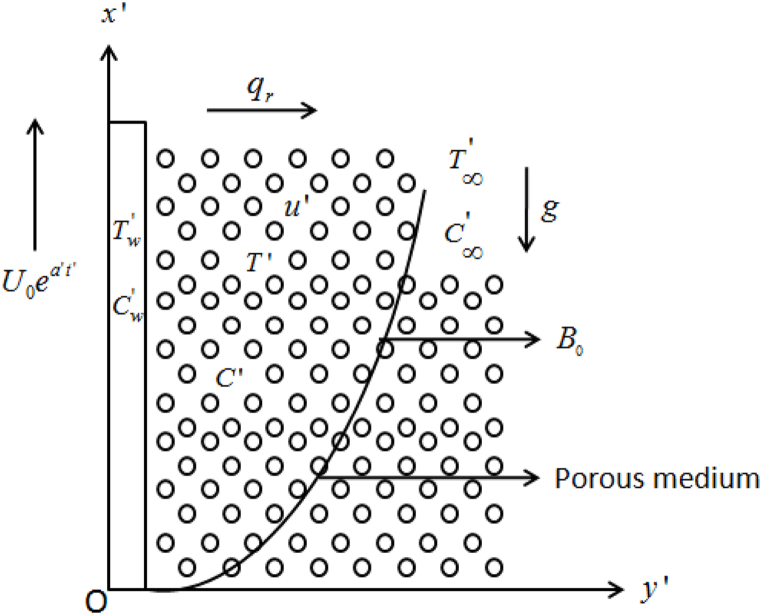
Geometric flow model.

Consider a mixed convection MHD heat generating viscous electrically conducting radiated Casson incompressible fluid flow from an exponentially accelerating vertical plate ingrained in a porous medium. The moment of the fluid is restrained to $y^{\prime }>0\text{,}$ where $y^{\prime }$ is the axis is selected normal to the surface of the plate, whilst the $x^{\prime }-$ axis is selected along with the plate into a vertical direction which is marked-out in Fig. 1. Initially, the plate, as well as fluid, stays restaging at a invariable concentration $C_{\infty }^{\prime }$ and temperature $T_{\infty }^{\prime }\text{.}$ Subsequently, when time $t^{\prime }>0\text{,}$ both the species concentration and temperature of the plate surface is either reduced or increased to $C_{\infty }^{\prime }+\left(C_{w}^{\prime }-C_{\infty }^{\prime }\right)t^{\prime }/t_{0}\text{,}$ and $T_{\infty }^{\prime }+\left(T_{w}^{\prime }-T_{\infty }^{\prime }\right)t^{\prime }/t_{0}\text{,}$ respectively for $t^{\prime }\leq t_{0}\text{,}$ which is after continued as constants $C_{w}^{\prime }$ and $T_{w}^{\prime }\text{,}$ respectively for $t^{\prime }>t_{0}\text{.}$ We made the following assumptions in the current study:

i. A transverse magnetic potency of $B_{0}$ is imposed along $y^{\prime }-$ axis and the 1st order chemical reaction is presumed within the fluid and dispersing species.
ii. The term viscous dissipation is incorporated into the conservation of energy equation.
iii. The effective magnetic force induced by the fluid flow is despised as the magnetic Reynolds' number is slender for liquefied-metals and partially ionized-fluid.
iv. The polarization of charges is deserted, because of no externally executed electric field.

By accepting the above interpretation, subjected to Boussinesq's approximation, the flow guiding equations of the physical phenomena amongst the applicable initial and boundary conditions derived as [5,8,10].

Continuity equation:

(1) $$\frac{\partial v^{\prime }}{\partial y^{\prime }}=0$$

Momentum equation:

(2) $$\frac{\partial u^{\prime }}{\partial t^{\prime }}=\left(1+\frac{1}{\beta }\right)\upsilon \frac{\partial^{2}u^{\prime }}{\partial y\prime^{2}}+g\beta^{T}\left(T^{\prime }-T_{\infty }^{\prime }\right)+g\beta^{C}\left(C\prime -C_{\infty }^{\prime }\right)-\frac{\upsilon \varphi }{K^{\prime }}\left(1+\frac{1}{\beta }\right)u^{\prime }-\frac{\sigma B_{0}^{2}}{\rho }u\prime \text{,}$$

Energy equation:

(3) $$\frac{\partial T^{\prime }}{\partial t^{\prime }}=\frac{k}{\rho C_{p}}\frac{\partial^{2}T^{\prime }}{\partial y\prime^{2}}-\frac{1}{\rho C_{p}}\frac{\partial q_{r}}{\partial y^{\prime }}+\frac{\upsilon }{C_{p}}\left(1+\frac{1}{\beta }\right){\left(\frac{\partial u^{\prime }}{\partial y^{\prime }}\right)}^{2}+\frac{\sigma B_{0}^{2}}{\rho C_{p}}{u^{\prime }}^{2}+\frac{\upsilon \varphi }{C_{p}K^{\prime }}\left(1+\frac{1}{\beta }\right){u^{\prime }}^{2}+\frac{Q_{G}}{\rho C_{p}}\left(T^{\prime }-T_{\infty }^{\prime }\right)\text{,}$$

Concentration equation:

(4) $$\frac{\partial C^{\prime }}{\partial t^{\prime }}=D_{CM}\frac{\partial^{2}C^{\prime }}{\partial y\prime^{2}}-k_{c}^{\prime }\left(C^{\prime }-C_{\infty }^{\prime }\right)\text{.}$$

and

(5a) $$u^{\prime }=0,T^{\prime }=T_{\infty }^{\prime },C'=C_{\infty }^{\prime }\;\text{for}\;y^{\prime }\geq 0\;\text{and}\;t'\leq 0\text{,}$$

(5b) $$u^{\prime }=u_{0}e^{\left(a't'\right)}\;\text{at}\;y^{\prime }=0\;\text{and}\;t\prime >0\text{,}$$

(5c) $$\left\{ \begin{array}{c} T^{\prime }=T_{\infty }^{\prime }+\left(T_{w}^{\prime }-T_{\infty }^{\prime }\right)t^{\prime }/t_{0}\;\text{at}\;y'=0\;\text{for}\;0<t'\leq t_{0},\\ T'=T_{w}^{\prime }\;\text{at}\;y'=0\;\text{for}\;t'>t_{0}\text{,} \end{array}\right.$$

(5d) $$\left\{ \begin{array}{c} C^{\prime }=C_{\infty }^{\prime }+\left(C_{w}^{\prime }-C_{\infty }^{\prime }\right)t^{\prime }/t_{0}\;\text{at}\;y'=0\;\text{for}\;0<t'\leq t_{0}\text{,}\\ C'=C_{w}^{\prime }\;\text{at}\;y'=0\;\text{for}\;t'>t_{0}\text{,} \end{array}\right.$$

(5e) $$u^{\prime }\to 0,T^{\prime }\to T_{\infty }^{\prime },C'\to C_{\infty }^{\prime }\;\text{as}\;y^{\prime }\to \infty \text{for}\;t'>0\text{.}$$

The radiation heat variability vector $q_{r}$ is analyzed with accepting the Rosseland approximate [15] of an optically thick grey-fluid, which is imparted by

(6) $$q_{r}=-\frac{4\sigma_{s}}{3k_{m}}\frac{\partial T\prime^{4}}{\partial y^{\prime }}$$

where $\sigma_{s}$ and $k_{m}\text{,}$ respectively represents the Stefan-Boltzmann and mean insertion constants. We simplify ${T^{\prime }}^{4}$ in Eq. (6) by implementing the Taylor's series expansion about $T_{\infty }^{\prime }$ and after overlooking the terms of greater order when the temperature distribution inside the flow is assumed as in Seth et al. [20], which implies

(7) $${T^{\prime }}^{4}≅4{T^{\prime }}_{\infty }^{3}T^{\prime }-3{T^{\prime }}_{\infty }^{4}$$

Using Eq. (6) into Eq. (5), we obtain

(8) $$\frac{\partial q_{r}}{\partial y^{\prime }}=-\frac{16\sigma_{s}T\prime_{\infty }^{3}}{3k_{m}}\frac{\partial^{2}T^{\prime }}{\partial y\prime^{2}}$$

Upon using Eq. (8) into Eq. (3), the energy equation becomes

(9) $$\frac{\partial T^{\prime }}{\partial t^{\prime }}=\left(\frac{k}{\rho C_{p}}+\frac{16\sigma_{s}T\prime_{\infty }^{3}}{3k_{m}\rho C_{p}}\right)\frac{\partial^{2}T^{\prime }}{\partial y\prime^{2}}+\frac{\upsilon }{C_{p}}\left(1+\frac{1}{\beta }\right){\left(\frac{\partial u^{\prime }}{\partial y^{\prime }}\right)}^{2}+\frac{\sigma B_{0}^{2}}{\rho C_{p}}{u^{\prime }}^{2}+\frac{\upsilon \varphi }{C_{p}K^{\prime }}\left(1+\frac{1}{\beta }\right){u^{\prime }}^{2}+\frac{Q_{G}}{\rho C_{p}}\left(T^{\prime }-T_{\infty }^{\prime }\right)$$

We preferred the complying dimensionless parameters and quantities;

$$\begin{array}{c} u=\frac{u^{\prime }}{u_{0}},\tau =\frac{t^{\prime }}{t_{0}},\eta =\frac{y^{\prime }}{t_{0}u_{0}},t_{0}=\frac{\upsilon }{u_{0}^{2}},S_{c}=\frac{\upsilon }{D_{CM}},P_{r}=\frac{\upsilon \rho C_{p}}{k},M^{2}=\frac{\sigma B_{0}^{2}\upsilon }{\rho u_{0}^{2}},a=\frac{a^{\prime }\upsilon }{u_{0}^{2}}\text{,}\\ \gamma =\frac{\upsilon k_{c}^{\prime }}{u_{0}^{2}},H=\frac{Q_{G}\upsilon }{u_{0}^{2}\rho C_{p}},N_{r}=\frac{16\sigma_{s}T\prime_{\infty }^{3}}{3kk_{m}},K=\frac{u_{0}^{2}K^{\prime }}{\upsilon^{2}\varphi },\theta =\frac{\left(T^{\prime }-T^{\prime }\right)}{\left(T_{w}^{\prime }-T_{\infty }^{\prime }\right)},\varphi =\frac{\left(C^{\prime }-C_{w}^{\prime }\right)}{\left(C_{w}^{\prime }-C_{\infty }^{\prime }\right)}\text{,}\\ G_{r}=\frac{g\beta^{T}\upsilon \left(T_{w}^{\prime }-T_{\infty }^{\prime }\right)}{u_{0}^{3}},G_{m}=\frac{g\beta^{C}\upsilon \left(C_{w}^{\prime }-C_{\infty }^{\prime }\right)}{u_{0}^{3}},E_{c}=\frac{u_{0}^{2}}{C_{p}\left(T_{w}^{\prime }-T_{\infty }^{\prime }\right)}\text{.} \end{array}\}$$

into Eqs. (2),(4),(8) and (5a)−(5e), we arrive at the following dimensionless version of the guiding PDEs with initial and boundary conditions of the flow problem:

(10) $$\frac{\partial u}{\partial \tau }=\left(1+\frac{1}{\beta }\right)\frac{\partial^{2}u}{\partial \eta^{2}}-\left[M^{2}+\left(1+\frac{1}{\beta }\right)\frac{1}{K}\right]u+G_{r}\theta +G_{m}\varphi \text{,}$$

(11) $$\frac{\partial \theta }{\partial \tau }=\frac{\left(1+N_{r}\right)}{P_{r}}\frac{\partial^{2}\theta }{\partial \eta^{2}}+E_{c}\left(1+\frac{1}{\beta }\right){\left(\frac{\partial u}{\partial \eta }\right)}^{2}+E_{c}\left[M^{2}+\frac{1}{K}\left(1+\frac{1}{\beta }\right)\right]u^{2}+H\theta \text{,}$$

(12) $$\frac{\partial \varphi }{\partial \tau }=\frac{1}{S_{c}}\frac{\partial^{2}\varphi }{\partial \eta^{2}}-\gamma \varphi \text{.}$$

Here $K,N_{r},M,a,H,\gamma ,E_{c},\beta ,\tau ,G_{r}$ and $G_{m}$ are, respectively, the permeability parameter, radiation parameter, magnetic intensity, acceleration parameter, heat-generated parameter, chemical reaction rate, Eckert number, Casson parameter, time and the thermal and solutal-Grashof numbers.

The initial and boundary conditions (5a)−(5e), becomes

(13a) u=0,θ=0,φ=0forη≥0andτ≤0,

(13b) $$u=e^{\left(a\tau \right)}\;at\;\eta =0\;\text{and}\;\tau >0\text{,}$$

(13c) $$\theta =\left\{ \begin{array}{c} \tau \;\text{at}\;\eta =0\;\text{for}\;0<\tau \leq 1,\\ 1\;\text{at}\;\eta =0\;\text{for}\;\tau >1, \end{array}\right.$$

(13d) $$\varphi =\left\{ \begin{array}{c} \tau \;\text{at}\;\eta =0\;\text{for}\;0<\tau \leq 1,\\ 1\;\text{at}\;\eta =0\;\text{for}\;\tau >1, \end{array}\right.$$

(13e) u→0,θ→0,φ→0asη→∞forτ>0.

The essential physical quantities of the described flow at the plate surface in the dimensionless version, the skin-friction $C_{f}=-\left(1+\frac{1}{\beta }\right){\left(\frac{\partial u}{\partial \eta }\right)}_{\eta =0}$, Nusselt number $N_{u}=-\left(1+N_{r}\right){\left(\frac{\partial \theta }{\partial \eta }\right)}_{\eta =0}$ and the Sherwood number $S_{h}=-{\left(\frac{\partial \varphi }{\partial \eta }\right)}_{\eta =0}$:

## 3 Computational procedure

Equations (10)−(12) are the system of coupled non-linear PDEs with relevant initial and boundary conditions Eq. (13a)−(13e) describing the flow pattern. The analytical (Exact) solutions to these equations are practically impossible. Hence, Eqs. (10)−(12) subjected to Eq. (13a)−(13e) are tackled numerically adopting the implicit Crank-Nicolson finite-difference procedure. This scheme is more accurate and efficient with improved stability characteristics. Therefore, implementing the numerical scheme on Eqs. (10)−(12), the resulting finite difference equations are:

(14) $$\left(\frac{u_{j}^{m+1}-u_{j}^{m}}{\Delta \tau }\right)=\left(1+\frac{1}{\beta }\right)\left(\frac{u_{j-1}^{m}-2u_{j}^{m}+u_{j+1}^{m}+u_{j-1}^{m+1}-2u_{j}^{m+1}+u_{j+1}^{m+1}}{2{\left(\Delta \eta \right)}^{2}}\right)-\left[M^{2}+\left(1+\frac{1}{\beta }\right)\left(\frac{1}{K}\right)\right]\left(\frac{u_{j}^{m}+u_{j}^{m+1}}{2}\right)+G_{r}\left(\frac{\theta_{j}^{m}+\theta_{j}^{m+1}}{2}\right)+G_{m}\left(\frac{\varphi_{j}^{m}+\varphi_{j}^{m+1}}{2}\right)$$

(15) $$\left(\frac{\theta_{j}^{m+1}-\theta_{j}^{m}}{\Delta \tau }\right)=\left(\frac{N_{r}+1}{P_{r}}\right)\left(\frac{\theta_{j-1}^{m}-2\theta_{j}^{m}+\theta_{j+1}^{m}+\theta_{j-1}^{m+1}-2\theta_{j}^{m+1}+\theta_{j+1}^{m+1}}{2{\left(\Delta \eta \right)}^{2}}\right)+H\left(\frac{\theta_{j}^{m}+\theta_{j}^{m+1}}{2}\right)+E_{c}\left(1+\frac{1}{\beta }\right){\left(\frac{u_{j+1}^{m}-u_{j}^{m}}{\Delta \eta }\right)}^{2}+E_{c}\left[M^{2}+\left(1+\frac{1}{\beta }\right)\left(\frac{1}{K}\right)\right]{\left(u_{j}^{m}\right)}^{2}$$

(16) $$\left(\frac{\varphi_{j}^{m+1}-\varphi_{j}^{m}}{\Delta \tau }\right)=\frac{1}{S_{c}}\left(\frac{\varphi_{j-1}^{m}-2\varphi_{j}^{m}+\varphi_{j+1}^{m}+\varphi_{j-1}^{m+1}-2\varphi_{j}^{m+1}+\varphi_{j+1}^{m+1}}{2{\left(\Delta \eta \right)}^{2}}\right)-\gamma \left(\frac{\varphi_{j}^{m}+\varphi_{j}^{m+1}}{2}\right)$$

The discretization of the initial and boundary conditions (13a)−(13e) results

(17a) u(j,0)=0,θ(j,0)=0,φ(j,0)=0forallj,

(17b) $$u\left(0,m\right)=e^{\left(a\tau \right)}\;f\text{or}\;\text{all}\;m\text{,}$$

(17c) $$\theta \left(0,m\right)=\left\{ \begin{array}{c} \tau \;\text{for}\;0<\tau \leq 1\;\text{and}\;\text{for}\;\text{all}\;m,\\ 1\;\text{for}\;\tau >1\;\text{and}\;\text{for}\;\text{all}\;m, \end{array}\right.$$

(17d) $$\varphi \left(0,m\right)=\left\{ \begin{array}{c} \tau \;\text{for}\;0<\tau \leq 1\;\text{and}\;\text{for}\;\text{all}\;m,\\ 1\;\text{for}\;\tau >1\;\text{and}\;\text{for}\;\text{all}\;m, \end{array}\right.$$

(17e) $$u\left(j_{\max},m\right)\to 0,\theta \left(j_{\max},m\right)\to 0,\varphi \left(j_{\max},m\right)\to 0\;\text{for}\;\text{all}\;m\text{.}$$

Where $j_{\max}$ correspond to ∞, the indices j and m corresponds to η and τ, Δη and Δτ are grid sizes in η and τ− directions, respectively. For the computational intent, the model's domain is considered a rectangle of finite dimensions. The boundary condition η→∞, has been fitted by $\eta_{\max}=2\text{,}$ which is more suitable to state free-stream flow characteristics in the presence of involvement of various embedded parameters. Eqs. (14)−(16) along with the use of Eq. (17a)−(17e) comprises a tri-diagonal format of equations at m− th level, which can be simplified by employing the Thomas algorithm. Our main concern is to fix the grid sizes along η and τ− directions, some numerical experimentation was done by first fixing the grid size Δτ=0.01, the finite difference code was run for different grid sizes Δη=0.01,0.05 and 0.001. The assumption of the dimensions of the rectangular domain are justified given that the boundary condition (17e) satisfied within the tolerance limit of $10^{-5}\text{.}$ It is found that no alteration in the numerical values of u,θ and φ, which confirms the solutions of u,θ and φ are grid-independent. Therefore, the grid sizes Δη=0.001 and Δτ=0.01 are considered for the entire numerical computation of the results. The local truncation error of the present numerical scheme is $O\left(\Delta \tau^{2}+\Delta \eta^{2}\right)\text{,}$ which turns to zero when Δτ and Δη tends to zero and also, which is unconditionally stable.

### 3.1 Code validation

Table 1 demonstrate the comparison of the skin-friction computed in this study by the Crank-Nicolson implicit finite difference technique in the absence of $N_{r},E_{c}$ and β=0 in the porosity term (Eq. (9)) with previously analytically computed results Kataria and Patel [10]. We confirm from this table that our results correlate very well with the results of Kataria and Patel [9], which establishes the accuracy of the utilized technique in this study. Further, it is concluded from Table 2 that our results in the limiting case i.e., β→∞ when $G_{m}=0,E_{c}=0,H=0,M=2,G_{r}=4,K=0.3\text{,}$ and $P_{r}=0.71$ constitute remarkable concordance with the results of Seth et al. [19], which supports our current work.

**Table 1:** The numerical outcomes correlation of the skin-friction $\left(C_{f}\right)$ for ramped and isothermal plate temperatures.

β	$G_{r}$	$G_{m}$	γ	M	K	H	τ	Ramped plate temperature		Isothermal plate temperature	
								Ref.	Present	Ref.	Present
0.5 0.6	3 4	4 5	5 5.2	2 2.2	0.8 0.9	−3 −2	0.4 0.5	9.0249 9.0894 9.0725 9.6241 8.9588 9.5520 8.9255 9.0331 10.5195	9.024828 9.089407 9.072536 9.624241 8.958801 9.552095 8.925500 9.033204 10.519309	5.7787 5.4333 5.6669 5.6860 5.7612 6.3676 5.6930 5.7429 6.3008	5.778757 5.433329 5.666747 5.686144 5.761212 6.367600 5.693081 5.742973 6.300794

**Table 2:** Comparison of the skin-friction $\left(C_{f}\right)$ in the limiting case for ramped and isothermal plate temperatures when $\beta \to \infty ,G_{m}=0,E_{c}=0,H=0,M=2,G_{r}=4,K=0.3\text{,}$ and $P_{r}=0.71$:

$N_{r}$	τ	Ramped temperature plate		Isothermal temperature plate	
		Seth et al. [19]	FDM results	Seth et al. [19]	FDM results
1 3 5	0.5 0.7 0.9 0.5 0.7 0.9 0.5 0.7 0.9	0.771220 0.863794 0.974772 0.774277 0.890407 1.018130 0.773208 0.899255 1.034050	0.771228 0.863801 0.974769 0.774275 0.890410 1.018156 0.773219 0.899298 1.034079	−0.214502 0.283120 0.775041 −0.204047 0.284840 0.775256 −0.197768 0.286484 0.775661	−0.214539 0.283154 0.775107 −0.204068 0.284901 0.775290 −0.197821 0.286493 0.775670

## 4 Discussion of the findings

To determine the physical aspects of the described model, the effects of assorted flow control parameters such as the radiation parameter, Casson parameter, plate acceleration parameter, thermal Grashof number, porosity parameter, magnetic parameter, Eckert number, heat propagation parameter, mass Grashof number, chemical reaction parameter and time on the velocity, temperature, concentration profiles of the Casson fluid, as well the wall-friction, Nusselt and Sherwood numbers in the two instances of ramped and isothermal plate temperatures are analyzed. Numerical estimations of the solutions of $u,\theta ,\varphi ,C_{f},N_{u}$ and $S_{h}$ were executed, and the output results were exhibited in graphical and tabular formats. The numerical computations were done by taking the default values of critical parameters as; $G_{r}=5,P_{r}=0.71\text{,}$ $K=1,M=5,\beta =0.5,G_{m}=5,N_{r}=2,S_{c}=0.66,H=2,E_{c}=0.3,\gamma =2,a=0.4$ and t=0.4, until otherwise clearly declared.

**Fig. 2:**
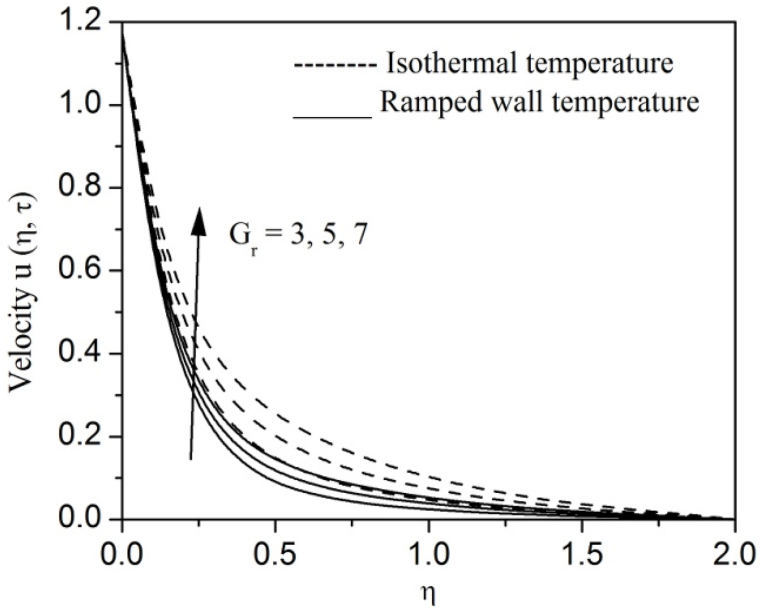
Velocity vs η for varying $G_{r}$:

**Fig. 3:**
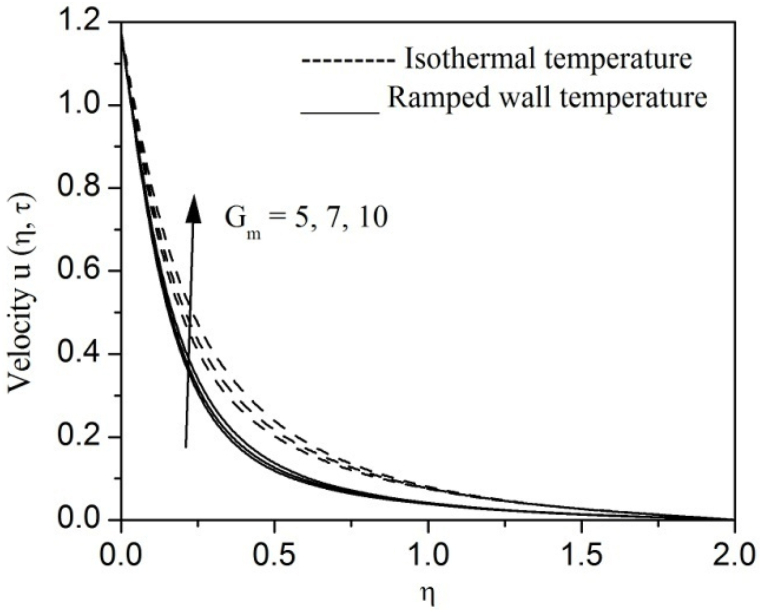
Velocity vs η for varying $G_{m}$:

**Fig. 4:**
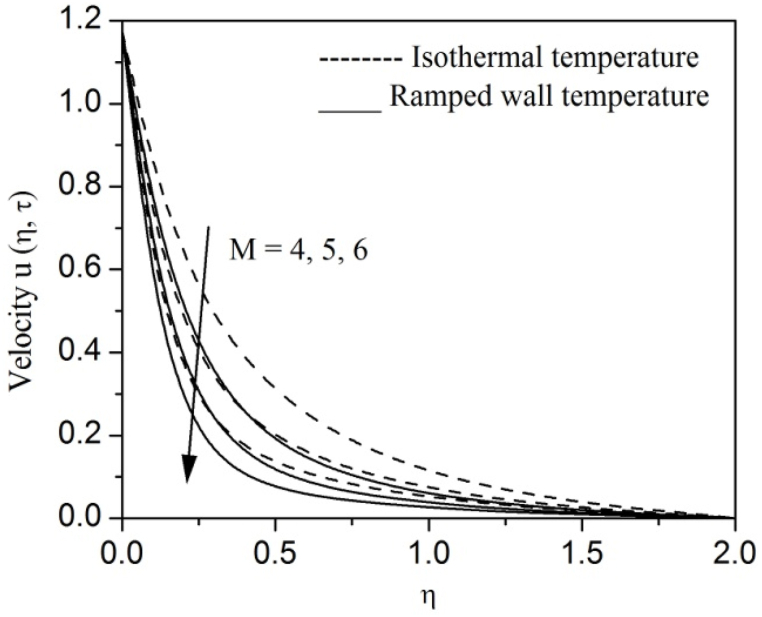
Velocity vs η for varying M:

**Fig. 5:**
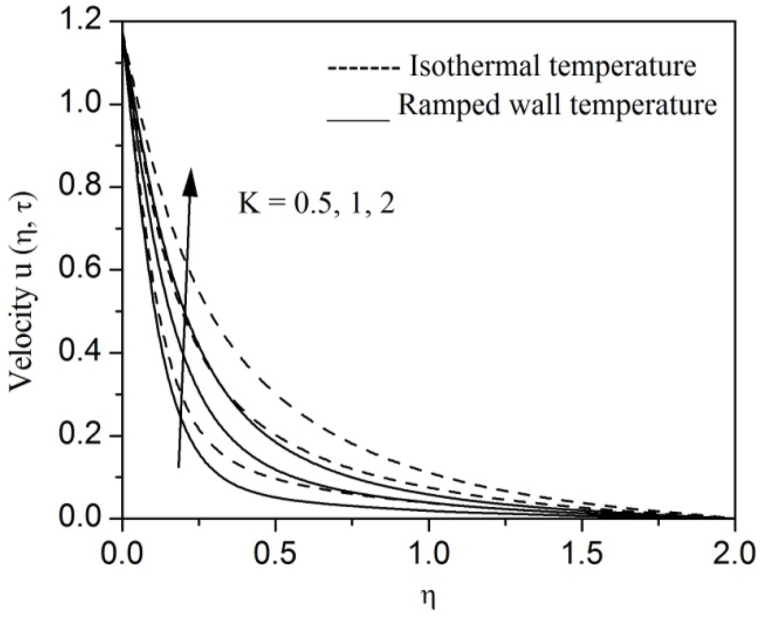
Velocity vs η for varying K:

**Fig. 6:**
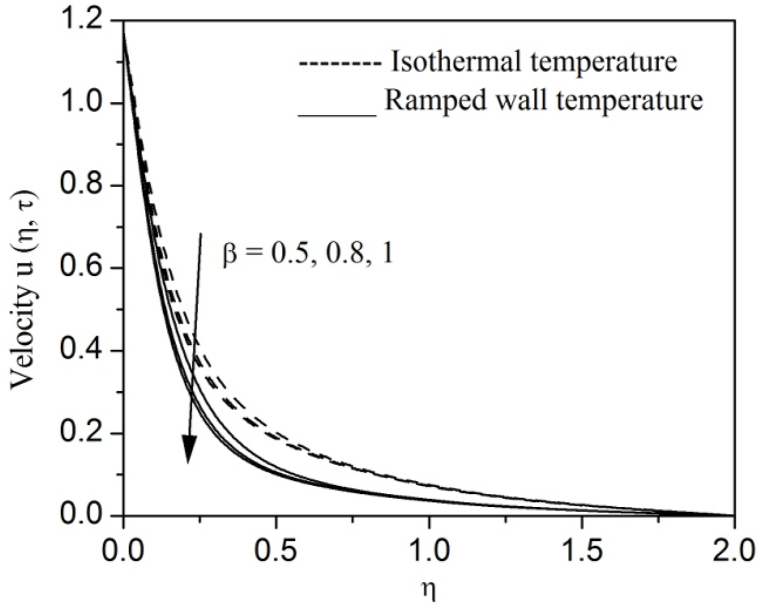
Velocity vs η for varying β:

**Fig. 7:**
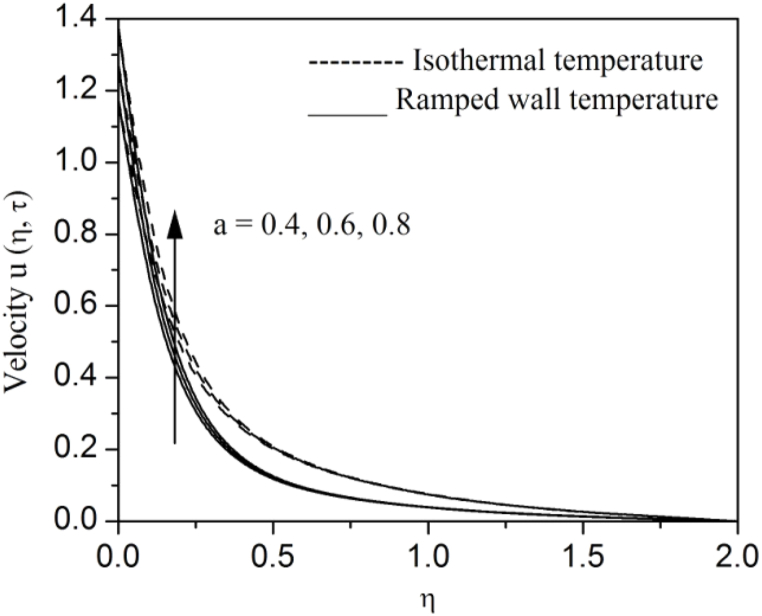
Velocity vs η for varying a:

**Fig. 8:**
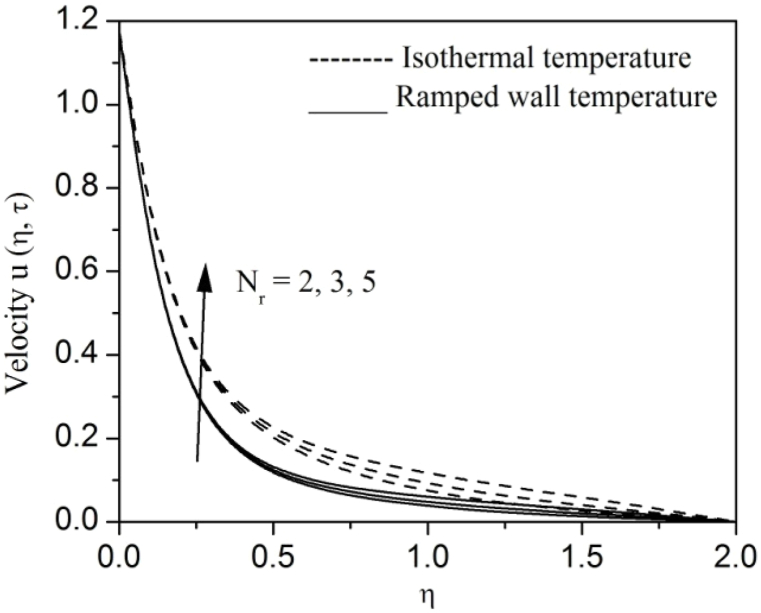
Velocity vs η for varying $N_{r}$:

**Fig. 9:**
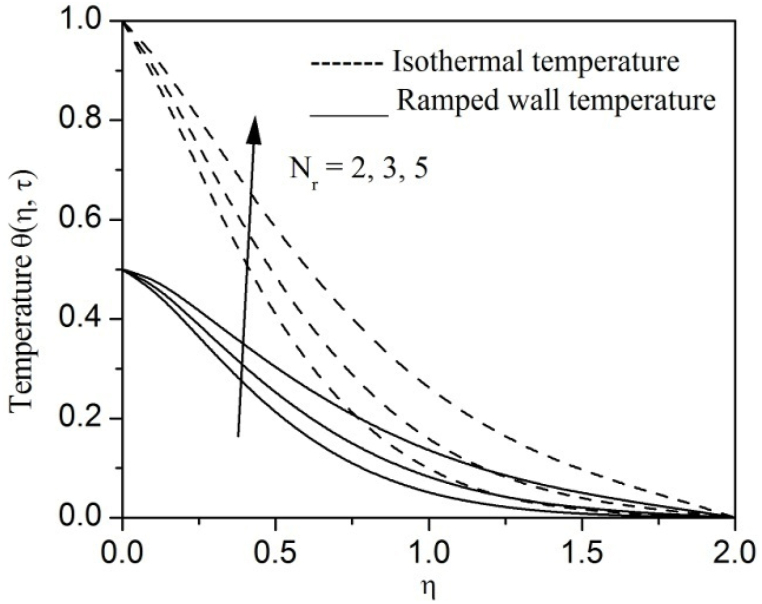
Temperature vs η for varying $N_{r}$:

**Fig. 10:**
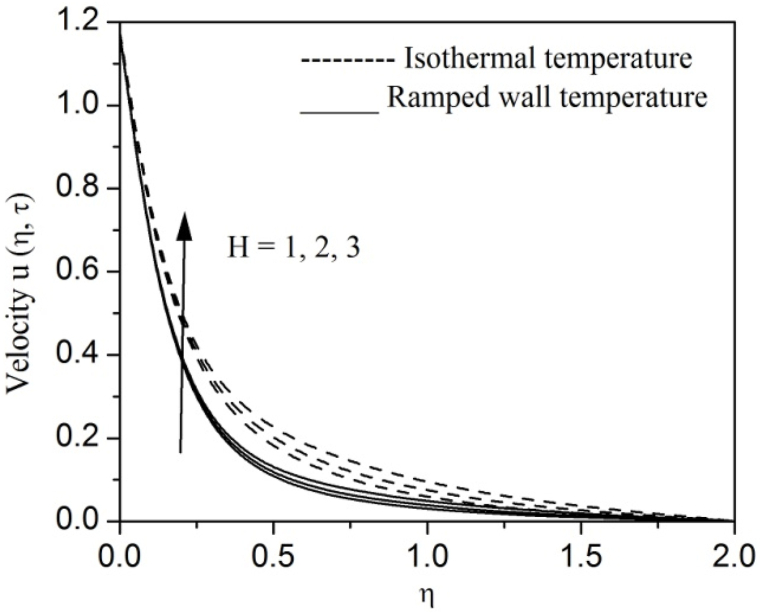
Velocity vs η for varying H:

**Fig. 11:**
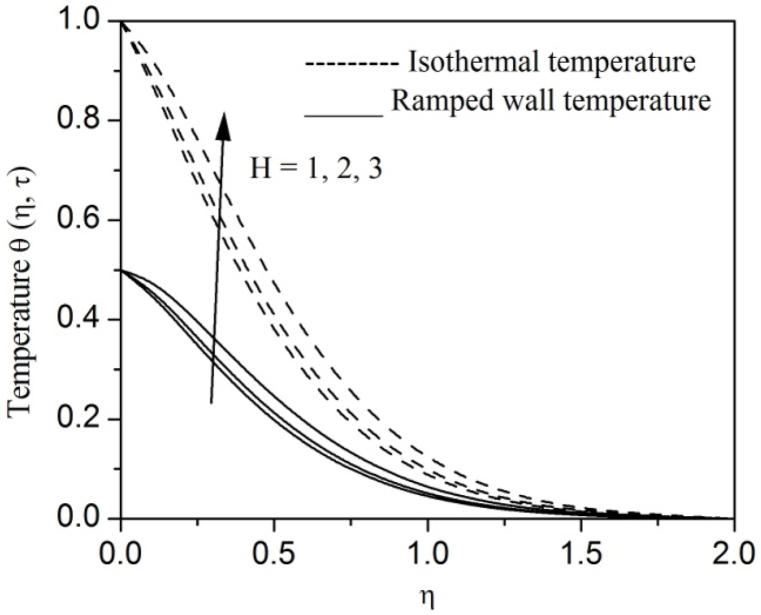
Temperature vs η for varying H:

**Fig. 12:**
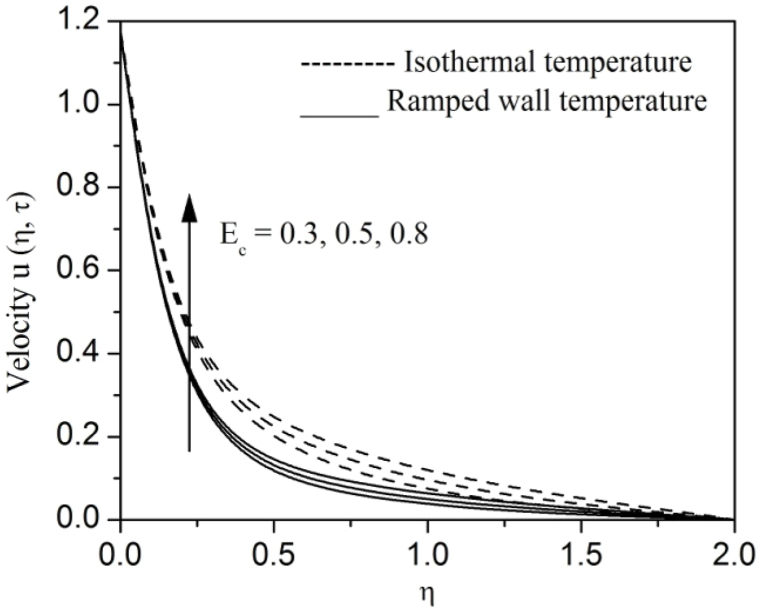
Velocity vs η for varying $E_{c}$:

**Fig. 13:**
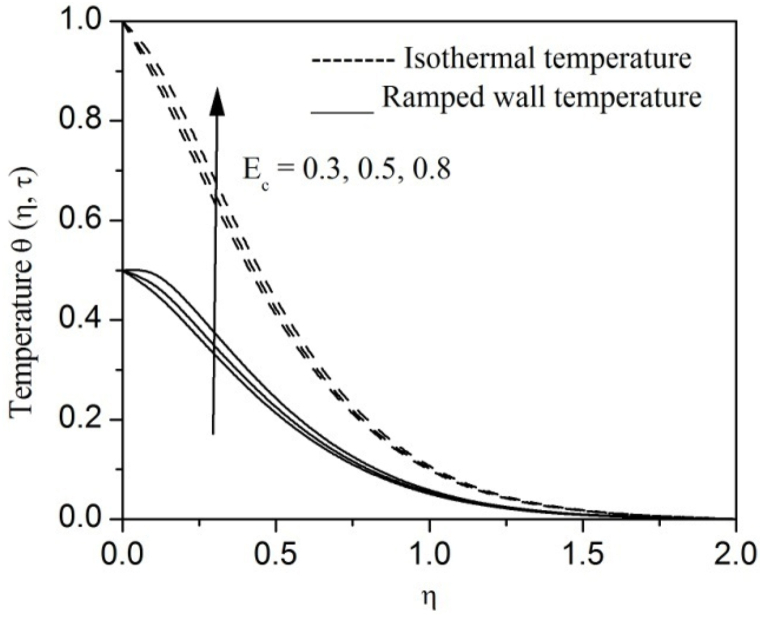
Temperature vs η for varying $E_{c}$:

**Fig. 14:**
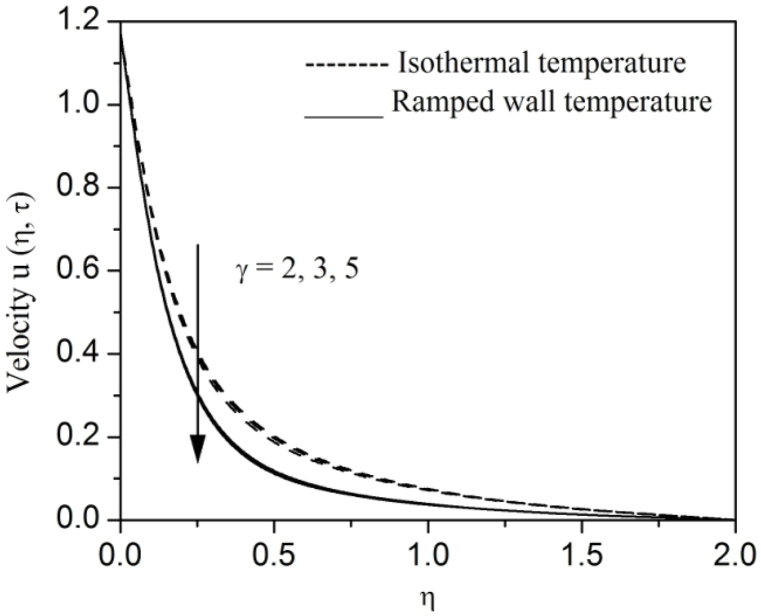
Velocity vs η for varying γ:

**Fig. 15:**
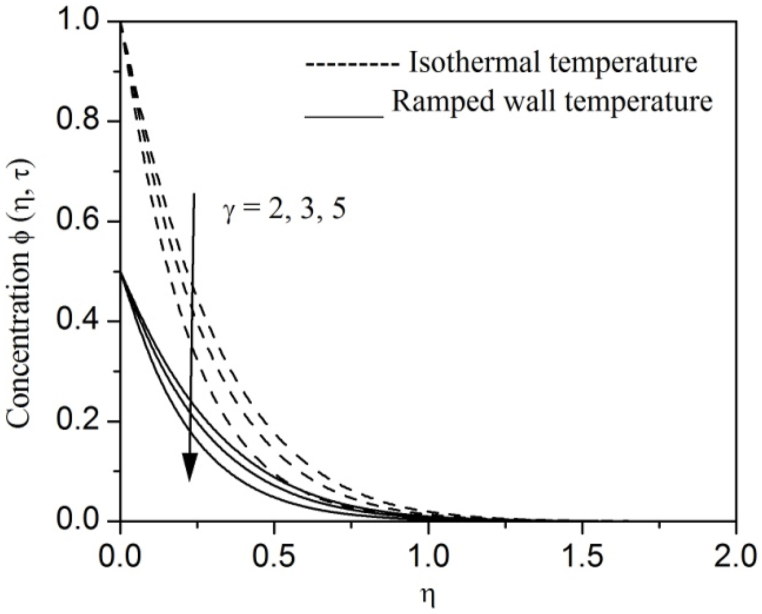
Concentration vs η for varying γ:

**Fig. 16:**
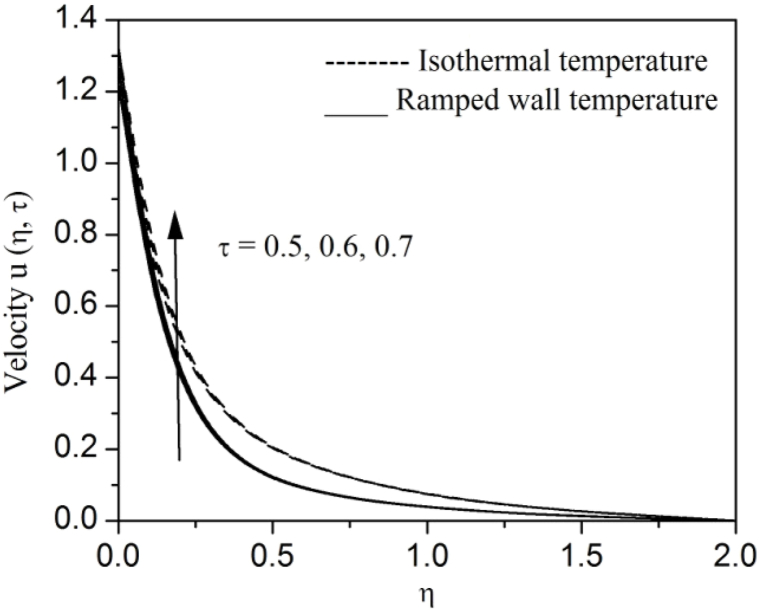
Velocity vs η for varying τ:

**Fig. 17:**
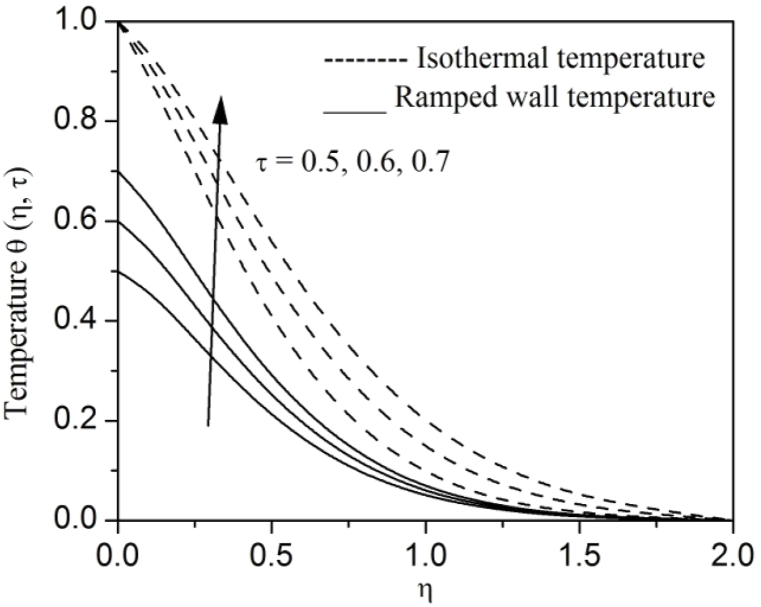
Temperature vs η for varying τ:

**Fig. 18:**
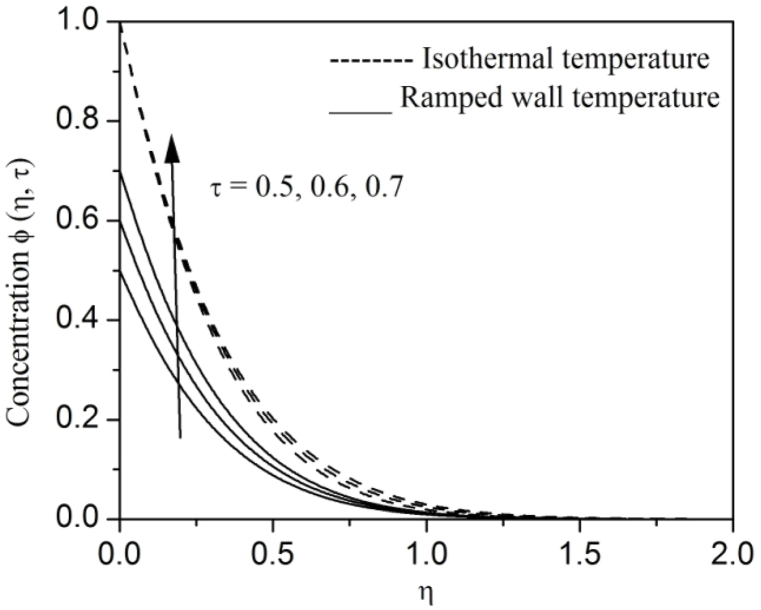
Concentration vs η for varying τ:

Fig. 2, Fig. 3, Fig. 4, Fig. 5, Fig. 6, Fig. 7, Fig. 8, Fig. 9, Fig. 10, Fig. 11, Fig. 12, Fig. 13, Fig. 14, Fig. 15, Fig. 16, Fig. 17, Fig. 18 are sketched to analyze the influent of $G_{r},G_{m},M,K,\beta ,a,N_{r},H,E_{c},\gamma$ and τ on Casson fluid velocity u, temperature θ and concentration φ against η for both ramp surface and isothermal temperature. Fig. 2, Fig. 3, respectively demonstrates the variation of the velocity distribution due to the alteration in the thermal and mass Grashof numbers $G_{r}$ and $G_{m}\text{.}$ It is remarked from these figures that the fluid movement speeds up with enlargement in $G_{r}$ and $G_{m}\text{,}$ which is physically reasonable. Since escalating values of $G_{r}$ and $G_{m}$ produce thermal and mass buoyancy effects that are strong as a consequence, fluid motion accelerates. The behavior of fluid velocity due to the implications of the magnetic field intensity M and permeability parameter K against η is described in Fig. 4, Fig. 5, respectively. A significant diminishing impact on the fluid movement with rising magnetic parameter is observed. In contrast, the opposite drift is recorded in the flow with mounting porosity parameter values. Physically, in an electrically conveying fluid, enforcement of magnetic force incites a delaying body effort well-known as the “Lorentz force” that can resist the fluid speed. The intensification K contributes to a diminution in the opposition of the porous medium; consequently velocity increases. Fig. 6, Fig. 7 drawn to perceive the influent of the Casson parameter β and plate acceleration parameter a on u against η, respectively. We observe a retarding impact on the flow with growing values of β whereas the reverse trend is identified with uprising values of a. Physically, increasing values of β, yield stress diminished; as a result, plastic dynamic viscosity improved and therefore fluid velocity gets reduced. Also, uprising values of a tends to have higher plate velocity; as a result, fluid moves faster adjacent to the plate. Fig. 8, Fig. 9 respectively, illuminate the variations of u and θ under the variation of radiation parameter $N_{r}$ against η. The increasing behavior is observed in both fluid speed and temperature with advancing values of $N_{r}\text{.}$ Because of the snowballing thermal radiation parameter gives a surplus means to scatter energy in the flow region, causing higher fluid temperature and consequently improving the fluid momentum. Fig. 10, Fig. 11 epitomize the repercussion of the heat-generating parameter H on the fluid velocity and temperature distributions, respectively. It is perceived that an increasing impact on both u and θ with growing heat generating parameter values. Physically, enhancement of heat generating parameter H creates additional heat in the flow, consequently enhancing the fluid's temperature and speeding up flow momentum. Fig. 12, Fig. 13, respectively, elucidate the impacts of viscous dissipation parameter $E_{c}$ on both velocity and temperature against η. One can see from these figures that both u and θ augmented on expanding values of $E_{c}\text{.}$ This is true because of larger values of $E_{c}$ implies that material particles become energetic due to the vast quantity of energy deposit, causing inflate temperature of the fluid, eventually intensify fluid velocity. The fallout of species chemical reaction γ on both velocity and concentration against η is emphasized in Fig. 14, Fig. 15, respectively. It is professed that there is a plummet in both u and φ with escalating γ, which is physically acceptable. Since an increase in γ gathering the collapse of species with top-rate results in a deflating effect on the fluid concentration, consequently, fluid flow lessens. It is ascertained from Fig. 16, Fig. 17, Fig. 18 that u,θ and φ increased with time progression. The behavior of u,θ and φ curves clearly agree with stated initial and boundary conditions. Very importantly, as a result of the ramped temperature condition, i.e., 0<t<1, fluctuating nature is identified in both temperature and concentration at the wall and the isothermal boundary condition, i.e., t>1, both temperature and concentration achieve its highest point and does not change. Further, from all Fig. 2, Fig. 3, Fig. 4, Fig. 5, Fig. 6, Fig. 7, Fig. 8, Fig. 9, Fig. 10, Fig. 11, Fig. 12, Fig. 13, Fig. 14, Fig. 15, Fig. 16, Fig. 17, Fig. 18, the identical nature is witnessed in the fluid velocity, temperature and concentration for ramp as well isothermal temperatures however, the magnitude of these flow fields dominating in the instance of isothermal temperature to that of ramp surface temperature.

The variation of $S_{h},N_{u}$ and $C_{f}$ under the dominance of $M,K,\beta ,N_{r},H,E_{c},\gamma ,a,G_{r},G_{m}\text{,}$ and τ are represented in Table 3, Table 4, Table 5 for both ramp and isothermal temperatures. Table 3 exposed that the influence of γ tends to improve $S_{h}$ for both thermal conditions. In the case of ramp surface temperature, $S_{h}$ ascend for time elapse, whilst the everted tendency is recorded in the case of isothermal plate temperature. Also, Table 3 shows a perfect accord between the results in this study and those of Kataria and Patel [10] and Seth et al. [20] obtained by exact solutions, which established the results obtained in this study are accurate. It is perceived from Table 4 that the value of $N_{u}$ upsurge with enlarged values of $N_{r},H$ and $E_{c}\text{.}$ Increasing time τ, in case of ramp surface temperature, $N_{u}$ strengthen whilst opposite tendency is noted for the isothermal plate temperature case. It is discovered from Table 5, that the value of skin friction escalates on enrichment in M,β,γ,a and τ values whilst overturn noticed with an expansion in $G_{r},G_{m},K,N_{r},H$ and $E_{c}$:

**Table 3:** A comparison study of $S_{h}$ when $S_{c}=0.22$:

t	γ	Sherwood number $S_{h}$					
		Ramp wall temperature			Isothermal temperature		
		Kataria and Patel [10]	Seth et al. [20]	FDM results	Kataria and Patel [10]	Seth et al. [20]	FDM Results
0.3 0.5 0.7 0.3 0.5 0.7 0.3 0.5 0.7	0.2 2 5	0.2956 0.3866 0.4632 0.3447 0.4881 0.6254 0.4169 0.6287 0.8389	0.295649 0.386593 0.463189 0.344659 0.488076 0.625355 0.416933 0.628694 0.838894	0.295649 0.386595 0.463188 0.344659 0.488076 0.625353 0.416930 0.628691 0.838893	0.5257 0.4284 0.3796 0.8399 0.7860 0.7579 1.1897 1.1294 1.0952	0.525702 0.428415 0.379505 0.839945 0.785973 0.757863 1.189700 1.129450 1.095220	0.525702 0.428415 0.379502 0.839944 0.785977 0.757861 1.189708 1.129455 1.095221

**Table 4:** Nusselt number $N_{u}$:

H	$N_{r}$	$E_{c}$	t	Nusselt number $N_{u}$	
				Ramped temperature	Isothermal temperature
2 1 3 2	2 3 5 2	0.3 0.5 0.8 0.3	0.4 0.5 0.6	0.473364 0.577416 0.249012 0.356352 0.220212 0.265572 0.193404 0.632952 0.792444	1.058650 1.226831 0.696070 0.871370 0.653507 0.890750 0.641130 0.790128 0.587630

**Table 5:** Skin-friction $C_{f}$:

$G_{r}$	$G_{m}$	M	K	β	$N_{r}$	H	$E_{c}$	γ	a	t	$C_{f}$	
											Ramped temperature	Isothermal temperature
5 3 7 5	5 7 10 5	5 4 6 5	1 0.5 1.5 1	0.5 0.8 1 0.5	2 3 5 2	2 1 3 2	0.3 0.5 0.8 0.3	2 3 5 2	0.4 0.6 0.8 0.4	0.4 0.6 0.7	2.137088 2.215188 2.059164 2.089828 2.018932 1.687992 2.521704 2.876060 1.727820 2.352308 2.443196 2.133184 2.128800 2.149820 2.122044 2.119656 2.100496 2.145060 2.158036 2.339604 2.558840 2.339604 2.447048	1.832812 1.985332 1.680552 1.738248 1.596368 1.308592 2.272656 2.671052 1.355520 2.007044 2.080484 1.825244 1.816756 1.857648 1.803464 1.804880 1.777220 1.848772 1.874736 2.035376 2.254660 2.035376 2.142844

## 5 Concluding remarks

In this work, the governing set of non-linear PDEs for the problem of radiating mixed convection MHD heat propagating reactive Casson dissipating fluid with ramp wall concentration and temperature travelling over an exponentially rushing vertical plate in a porous backdrop including Joule heating effect are examined numerically. the adequate knowledge of mixed convection MHD heat propagating reactive-radiative Casson dissipating flow is significant in the perspective of space technology, lubrication, tribology, pharmaceutical and medical industries and processes forcing high temperatures such as glass production, furnace technology and fire dynamics. Noteworthy outcomes of the analysis are.

(i) For two thermal conditions, the fluid velocity exacerbates for larger values of $G_{r},G_{m},K,N_{r},H$ and $E_{c}$ but reversal trend was observed in the fluid velocity for expanding values of M,β and γ. The skin-friction established exactly opposite version to that of fluid velocity with these parameters.
(ii) The fluid velocity escalates on widening values of a and τ, for two thermal conditions. The skin-friction is also showed the same nature with these parameters.
(iii) The temperature distribution expands with advancing $N_{r},H$ and $E_{c}$ values for two thermal conditions but Nusselt number expressed contrary demeanor to the temperature with these parameters.
(iv) For two thermal conditions, rising γ values caused to condense the concentration distribution but Sherwood number showed opposing trend to the concentration with γ:
(v) Both Nusselt and Sherwood numbers upsurges with increasing τ for ramp temperature but a reverse propensity was exhibited for isothermal temperature.
(vi) Importantly, the similar feature was endorsed in all the three flow fields for ramp as well isothermal temperatures although, in the sense of the magnitude, all three flow fields predominates for isothermal temperature to that of ramp surface temperature.

**Future suggestions:** The researchers may extend the current study.

(i) To other non-Newtonian fluid models such as Walter's-B fluid, second-grade fluid, Jeffery fluid, Maxwell fluid and Oldroyd-B fluid.
(ii) It's a brilliant idea to include the significances thermo-diffusion and diffusion-thermo in the governing equations.
(iii) To different nanofluid models for different applications.

## CRediT authorship contribution statement

**B. Prabhakar Reddy:** Writing – original draft, Validation, Methodology, Conceptualization. **P.M. Matao:** Validation, Methodology, Investigation, Formal analysis, Conceptualization. **J.M. Sunzu:** Writing – review & editing, Visualization, Supervision, Investigation, Formal analysis.

## Declaration of competing interest

The authors declare that they have no known competing financial interests or personal relationships that could have appeared to influence the work reported in this paper.

## Nomenclature

$u^{\prime }-$: Velocity along $x^{\prime }-$ axis $\left(ms^{-1}\right)$
k−: Thermal conductivity $\left(Wm^{-1}K^{-1}\right)$
M−: Magnetic field parameter
H−: Heat generation parameter (K)
$q_{r}-$: Radiative heat flux $\left(Wm^{-2}\right)$
$C^{\prime }-$: Fluid concentration $\left(molm^{-3}\right)$
$P_{r}-$: Prandtl number
θ−: Dimensionless temperature (K)
$C_{\infty }^{\prime }-$: Free stream concentration $\left(molm^{-3}\right)$
$E_{c}-$: Eckert number
u−: Dimensionless velocity $\left(ms^{-1}\right)$
$a^{\prime }-$: Plate acceleration parameter $\left(ms^{-1}\right)$
$\beta^{T}-$: Coefficient of thermal expansion $\left(K^{-1}\right)$
K−: Porosity parameter
$Q_{G}-$: Heat generation coefficient
$T^{\prime }-$: Fluid temperature (K)
υ−: Kinematic viscosity $\left(m^{2}s^{-1}\right)$
$S_{c}-$: Schmidt number
$\beta^{C}-$: Coefficient of solute expansion $\left(K^{-1}\right)$
$G_{m}-$: Mass Grashof number
$T_{w}^{\prime }-$: Temperature at the wall (K)
$T_{\infty }^{\prime }-$: Free stream temperature (K)
g−: Acceleration on account of gravity $\left(ms^{-2}\right)$
$k_{C}^{\prime }-$: Chemical reaction coefficient
φ−: Dimensionless concentration
β−: Casson parameter
$C_{w}^{\prime }-$: Concentration at the wall $\left(molm^{-3}\right)$
$G_{r}-$: Thermal Grashof number
φ−: Permeability parameter
ρ−: Fluid density $\left(Kgm^{-3}\right)$
σ−: Electrical conductivity $\left(Sm^{-1}\right)$
$D_{CM}-$: Mass diffusivity $\left(m^{2}s^{-1}\right)$
$B_{0}-$: Uniform magnetic field strength $\left(Am^{-1}\right)$
$t^{\prime }-$: Time (s)
$C_{p}-$: Specific heat at a constant pressure $\left(Jkg^{-1}k\right)$
$N_{r}-$: Radiation parameter
a−: Dimensionless acceleration parameter $\left(ms^{-1}\right)$
$C_{f}-$: Skin-friction $\left(Nm^{-2}\right)$
$N_{u}-$: Nusselt number
$S_{h}-$: Sherwood number
